# The impact of cohort inclusion/exclusion criteria on pregnancy weight gain chart percentiles

**DOI:** 10.1017/S0007114524001855

**Published:** 2024-09-28

**Authors:** Thais Rangel Bousquet Carrilho, Lisa M. Bodnar, Kari Johansson, Gilberto Kac, Jennifer A. Hutcheon

**Affiliations:** 1 Department of Obstetrics and Gynaecology, Faculty of Medicine, University of British Columbia, Vancouver, Canada; 2 Department of Epidemiology, School of Public Health, University of Pittsburgh, Pittsburgh, USA; 3 Clinical Epidemiology Division, Department of Medicine Solna, Karolinska Institutet, Stockholm, Sweden; 4 Division of Obstetrics, Department of Women’s Health, Karolinska University Hospital, Stockholm, Sweden; 5 Nutritional Epidemiology Observatory, Josué de Castro Nutrition Institute, Federal University of Rio de Janeiro, Rio de Janeiro, Brazil

**Keywords:** Gestational weight gain, Reference, Standards, Pregnancy

## Abstract

Pregnancy weight gain standards are charts describing percentiles of weight gain among participants with no risk factors that could adversely affect weight gain. This detailed information is burdensome to collect. We investigated the extent to which exclusion of various pre-pregnancy, pregnancy and postpartum factors impacted the values of pregnancy weight gain percentiles. We examined pregnancy weight gain (kg) among 3178 participants of the US nuMoM2b-Heart Health Study (HHS). We identified five groups of potential exclusion criteria for pregnancy weight gain standards: socio-economic characteristics (group 1), maternal morbidities (group 2), lifestyle/behaviour factors (group 3), adverse neonatal outcomes (group 4) and longer-term adverse outcomes (group 5). We established the impact of different exclusion criteria by comparing the median, 25th and 75th percentiles of weight gain in the full cohort with the values after applying each of the five exclusion criteria groups. Differences > 0·75 kg were considered meaningful. Excluding participants with group 1, 2, 3 or 4 exclusion criteria had no impact on the 25th, median or 75th percentiles of pregnancy weight gain. Percentiles were only meaningfully different after excluding participants in group 5 (longer-term adverse outcomes), which shifted the upper end of the weight gain distribution to lower values (e.g. 75th percentile decreased from 19·6 kg to 17·8 kg). This shift was due to exclusion of participants with excess postpartum weight retention > 5 kg or > 10 kg. Except for excess postpartum weight retention, most potential exclusion criteria for pregnancy weight gain standards did not meaningfully impact chart percentiles.

Gestational weight gain (GWG) is an important indicator of health during pregnancy. Sub-optimal weight gain has been linked with adverse outcomes for both mother and child, including small- and large-for-gestational age birth, gestational diabetes and pre-eclampsia^(1–3)^. Interventions during pregnancy to optimise weight gain have been shown to improve several of these outcomes^(4)^, highlighting the importance of monitoring weight gain in antenatal care.

Charts to monitor GWG can be standards (prescriptive charts of how weight gain ought to be) or references (descriptive charts of what weight gain was like in an unselected population)^(5,6)^. From a theoretical perspective, standards are preferable for identifying individuals at risk of insufficient or excessive weight gain in clinical practice^(5)^. Yet, from a practical perspective, standards are more challenging to create because they require detailed information on all conditions that can adversely affect pregnancy weight gain, such as maternal diet, physical activity^(7)^ and adverse postpartum outcomes such as excess postpartum weight retention and child overweight/obesity. This detailed information is often not available in cohorts used to create such charts: none of the nine pregnancy weight gain charts created in the past 10 years have incorporated diet, exercise or postpartum outcomes into their exclusion criteria^(8–16)^. Most studies excluded individuals with hypertension, diabetes, hypertensive disorders of pregnancy and/or gestational diabetes^(8–15)^. Adverse neonatal outcomes, such as the birth of small-/large-for-gestational age infants, with low birth weight, macrosomia or with congenital abnormalities, were excluded in about half of the charts^(9,13–15)^.

Collection of this detailed information can be burdensome and is only warranted if the exclusion of individuals with the adverse conditions of interest will meaningfully impact the percentile values of the weight gain chart. Identifying which exclusion criteria meaningfully impact those percentiles will support efficient use of resources in the design of new pregnancy weight gain standards and help to identify the extent to which the utility of existing charts is decreased by the lack of information on many adverse lifestyle factors. Thus, we aimed to identify how normative values of pregnancy weight gain are affected by the exclusion of individuals with pre-pregnancy, pregnancy and postpartum factors adversely linked with pregnancy weight gain.

## Methods

### Study sample

We used data from the nuMoM2b-Heart Health Study (HHS), a 2–5-year follow-up study of the Nulliparous Pregnancy Outcomes Study: monitoring mothers-to-be (nuMoM2b) cohort. Briefly, the nuMoM2b was a cohort study following nulliparous pregnant women and other nulliparous pregnant individuals in eight US clinical centres, with the recruitment occurring from 2010 to 2013. Participants were recruited from 6 weeks + 0 d to 13 weeks + 6 d of pregnancy if they had a viable singleton pregnancy and no previous pregnancy lasting 20 weeks or more. Study visits occurred in each pregnancy trimester. At delivery, chart abstraction was performed by trained research team members^(17)^. The HHS followed participating individuals after delivery, with continuous monitoring of eligible participants through telephone interviews and an in-person visit up to 7 years after delivery (median 3·2 years). In this in-person visit, information on psychosocial and medical characteristics was collected through questionnaires. Clinical measurements and biological specimens were also obtained. A detailed protocol of the HHS is available elsewhere^(18)^.

For this study, we limited our analysis to HHS participants who did not have a stillbirth or neonatal death in their nuMoM2b pregnancy, had an in-person visit from 2 to 5 years postpartum and had complete data for our key variables of interest. The dataset of individuals meeting these minimum inclusion criteria is referred to as the ‘full cohort’ in this study.

### Weight and weight gain measurements

Weight gain during pregnancy (kg) was calculated as the difference between the weight measured in the study visits and self-reported pre-pregnancy weight. Four weight gain measurements were available for this study (at 1st, 2nd, 3rd trimesters and at delivery). Weight gain measurements > + 6 or < −6 sd for gestational age^(8,10)^ were considered outliers and removed from the analysis. This conservative cut-off was chosen to minimise the loss of weight gain values that are extreme, but still plausible.

We selected specific windows for comparison, considering the available sample size in each gestational week: first visit weight gain (measurements obtained between 11 and 14 weeks); second visit weight gain (measurements from 27 to 30 weeks) and at delivery (measurements obtained between 26 and 42 weeks). The broader range of gestational ages for weight gain at delivery enabled us to examine the impact of excluding individuals with adverse perinatal outcomes such as preterm birth, which would not have been possible if we limited total weight gain to a narrower window (e.g. term only). However, because the window of gestational ages in which delivery happened varied from 26 to 42 weeks, we also compared *z* scores of weight gain at delivery according to gestational age and pre-pregnancy BMI^(8,10)^.

Maternal pre-pregnancy BMI (kg/m^2^) was calculated using self-reported pre-pregnancy weight and maternal height obtained in the first study visit. BMI was classified according to the WHO cut-offs: underweight, BMI < 18·5 kg/m^2^; normal weight, BMI ≥ 18·5 and < 25·0 kg/m^2^; overweight, BMI ≥ 25·0 and < 30·0 kg/m^2^; and obesity, BMI ≥ 30 kg/m^2(19)^.

### Pre-pregnancy, pregnancy and postpartum exclusion criteria

We identified pre-pregnancy, pregnancy and postpartum factors available in the HHS dataset that could be considered as exclusion criteria when creating a pregnancy weight gain standard based on the 2009 Institute of Medicine report’s chapter on weight gain determinants^(7)^ and on adverse pregnancy outcomes related to weight gain^(20)^ (Table 1). We additionally included information related to sleep quality, as recent work suggests this may be a determinant of weight gain^(36)^. The criteria were classified into five groups of associated characteristics for parsimony: Group 1 – Socio-economic and demographic characteristics; 2 – Pre-existing co-morbidities and maternal pregnancy complications; 3 – Maternal lifestyle and behaviour factors; 4 – Adverse neonatal outcomes; and 5 – Longer-term postpartum outcomes.

**Table 1.** Pre-pregnancy, pregnancy and postpartum exclusion criteria

Group	Exclusion criteria	Definition/source
1 – Socio-economic and demographic characteristics	Maternal age	Age ≤ 19 and ≥ 40 years old at the first study visit (between 6 + 0 and 13 + 6 weeks)
	Low maternal education	Completion of ≤ High School graduation
	Low socio-economic status	Proxy: Maternal public/self-pay health insurance
2 – Pre-existing co-morbidities and maternal pregnancy complications	Pre-pregnancy hypertension	Classified using variables on signs and symptoms abstracted from the medical record and adjudicated via chart review
	Diabetes	Classified using laboratory test results abstracted from the medical records and adjudicated via chart review
	Gestational diabetes	Classified using laboratory test results abstracted from the medical record and adjudicated via chart review. The 2018 ACOG diagnostic criteria was adapted and applied^(21)^
	Pre-eclampsia	Classified using signs and symptoms abstracted from the medical record and adjudicated via chart review. The 2013 ACOG criteria was adapted and applied^(22)^
	Unplanned caesarean section	Caesarean delivery occurring after the onset of labour
	Severe first-trimester nausea and vomiting	Pregnancy-Unique Quantification of Emesis and Nausea Index^(23)^ of 6 or more
	Other diseases	Conditions that could either affect weight during pregnancy or are treated with medications that could affect weight, abstracted from the medical record and adjudicated via chart review. Conditions included anaemia, hypo/hyperthyroidism, heart diseases, kidney diseases, sickle cell disease, bleeding disorders, autoimmune diseases, mental health conditions, gynaecological conditions or sexually transmitted infections
3 – Maternal lifestyle and behaviour factors	Smoking during pregnancy	Obtained in the study questionnaire in the first visit, classified as yes/no
	Early-pregnancy binge drinking	Any single occasion of drinking four or more drinks in the previous month, obtained in the first study visit
	Poor diet quality	Lowest tercile of the Healthy Eating Index 2015 obtained from food consumption data from a validated semi-quantitative FFQ^(24,25)^
	Low physical activity in the first pregnancy trimester	< 450 MET h/week obtained from a physical activity log referring to the previous 4 weeks obtained from the study questionnaire in the first study visit^(26)^
	High first-trimester perceived stress	Score of 27–40 on the perceived stress scale obtained from the study questionnaire in the first study visit^(27)^
	High first-trimester maternal anxiety	Score of 60–80 on the State-Trait Anxiety Inventory obtained from the study questionnaire in the first study visit^(28)^
	First-trimester depressive symptoms	Score ≥ 11 on the Edinburgh Postnatal Depression Scale obtained from the study questionnaire in the first study visit^(29)^
	Poor sleep quality	Individuals who reported their sleeping as ‘restless’ in the previous 4 months in the sleep questionnaire obtained in the first study visit
4 – Adverse neonatal outcomes	Preterm birth	Gestational age at birth < 37 weeks^(30)^
	Small-for-gestational-age (SGA)	Birth weight for gestational age < 10th percentile^(31)^
	Large-for-gestational-age (LGA)	Birth weight for gestational age > 90th percentile^(31)^
	Low birth weight	Birth weight < 2500 g
	Macrosomia	Birth weight > 4000 g
5 – Longer-term postpartum outcomes	Excess postpartum weight retention	Calculated as the difference between weight at the 2–5-year postpartum visit and self-reported pre-pregnancy weight and defined using two cut-offs: > +5 kg^(32)^ and > +10 kg.
	Child overweight or obesity	Weight-for-age ≥ 85th percentile of the CDC charts^(33)^
	Maternal metabolic syndrome	Presence of least three of the following conditions: (1) waist circumference ≥ 88 cm, (2) systolic blood pressure ≥ 130 mmHg and/or diastolic blood pressure ≥ 85 mmHg or antihypertensive drug treatment for a history of hypertension, (3) fasting glucose ≥ 100 mg/dl, (4) TAG ≥ 150 mg/dl or drug treatment for elevated TAG or (5) HDL-cholesterol < 50 mg/dl or drug treatment for low HDL-cholesterol^(34,35)^

### Statistical analyses

We used absolute (*n*) and relative (%) frequencies to describe the characteristics of study participants. We established the impact of different exclusion criteria for a weight gain standard by comparing the median, 25th and 75th percentiles of weight gain in the full cohort with the median, 25th and 75th percentiles obtained after applying each of the five groups of exclusion criteria. We removed all characteristics in each group together for ease of presentation but planned a priori to evaluate the impact of individual exclusion criteria if meaningful differences in weight percentiles were observed after excluding the group they were in. We did not explore more extreme percentiles (e.g. 5th or 10th percentiles) due to the small sample sizes remaining after applying many exclusion criteria.

A group of variables was considered to have a meaningful impact on weight gain if the difference between the median, 25th or 75th percentiles of the full cohort and the sub-cohort excluding that group was > 0·75 kg. This value was considered to be meaningful from a clinical/public health perspective, as systematic reviews and meta-analysis of pregnancy weight gain interventions have shown that the mean weight gain changes of this amount are not associated with a significant reduction in the risks of maternal and infant adverse outcomes^(4,37)^. For the *z* scores of weight gain at delivery, differences of 0·15 *z* score were considered meaningful. This value corresponds to a 0·75 kg change in weight gain at 40 weeks based on charts created for normal-weight US women^(8)^.

For these meaningful variables, 95 % CI for the median, 25th and 75th percentiles of GWG were calculated using quantile regressions. We examined the distribution of weight gain at delivery for our primary analysis, and weight gain at the 1st and 2nd trimester visits in secondary analyses. We also constructed density plots for GWG at delivery in the full cohort and in the sub-cohorts created after the exclusion of individuals with an adverse characteristic in each group. These analyses were conducted in the complete dataset. We conducted a sensitivity analysis in which we stratified by pre-pregnancy BMI (participants with normal weight, overweight and obesity only). Our decision to combine all BMI groups in our primary analysis was based on the relatively small number of participants with overweight and obesity after exclusions.

## Results

The initial sample of the HHS study included 4405 participants. Excluding nineteen stillbirth/neonatal deaths and 1208 individuals with missing data for our key variables left 3178 participants for analysis (online Supplementary Fig. 1). The proportion of missing data for each of the key characteristics is shown in online Supplementary Table S1. The variables with the highest degree of missing values were diet quality (15 %), sleep quality (11 %) and maternal anxiety (10 %). There were no meaningful differences in the distribution of weight gain at delivery between individuals excluded due to missing data and those retained in our analytic cohort (online Supplementary Fig. 2).

In the full cohort, 114 (3·6 %) participants were classified with underweight, 1753 (55 %) with normal weight, 670 (21 %) with overweight and 641 (20 %) with obesity. The majority of individuals with obesity (52·2 %) were classified in class 1 (BMI ≥ 30·0 and < 35·0 kg/m^2^). Only fifty (1·6 %) participants had experienced weight loss at delivery (data not shown in tables). As shown in Table 2, most participants had been to college (85 %) and had private health insurance (75 %). Nine (9 %) per cent of participants developed pre-eclampsia, 20 % had an unplanned caesarean delivery and 44 % had another disease during pregnancy, the most common of which was anaemia (approximately 11 %). Most participants (54 %) were sedentary (practiced < 450 MET h/week of physical activity) and had a diet quality score under 70 of 100 possible points (66 %). Twelve per cent experienced depression, while 19 % described their sleep in the previous four weeks as restless.

**Table 2.** Description of the sample according to the selected groups of exclusion criteria (*n* 3178 individuals from the Heart Health Study) (Numbers and percentages; median values and interquartile ranges)

Categorical variables	*n*	%	GWG at delivery (kg)	
			Median	Interquartile range
Group 1 – Socio-economic and demographic variables				
Maternal age (years)				
Adolescent (≤ 19·0)^*^	228	7·2	14·5	10·9, 20·5
Adults (19·1–39)	2893	91·0	15·4	11·8, 19·5
Older (> 39)^*^	57	1·8	14·5	10·1, 18·5
Maternal education				
≤ High School Grad^*^	468	14·7	14·5	10·2, 20·0
Some College/Associates	930	29·3	15·4	11·3, 20·0
Bachelor’s degree	993	31·2	15·7	12·2, 19·2
Graduate degree	787	24·8	15·0	12·3, 18·7
Public health insurance				
Private	2372	74·6	15·5	12·3, 19·3
Public/self-pay^*^	806	25·4	14·8	10·0, 20·0
Group 2 – Pre-existing co-morbidities and maternal pregnancy complications				
Pre-pregnancy hypertension				
No	3055	96·1	15·5	11·8, 19·5
Yes^*^	123	3·9	14·0	8·1, 18·0
Pre-eclampsia^†^				
No	2892	91·0	15·0	11·6, 19·2
Yes^*^	286	9·0	16·4	12·3, 20·9
Pre-pregnancy diabetes				
No	3138	98·7	15·4	11·8, 19·5
Yes^*^	40	1·3	14·0	10·3, 17·3
Gestational diabetes^‡^				
No	3030	95·3	15·5	11·8, 19·5
Yes^*^	148	4·7	12·7	8·2, 17·8
Other diseases^§^				
No	1770	55·7	15·3	11·6, 19·5
Yes^*^	1408	44·3	15·3	11·8, 19·5
Unplanned caesarean section				
No	2536	79·8	15·0	11·5, 19·1
Yes^*^	642	20·2	16·4	12·1, 20·9
Severity of nausea and vomiting in the first trimester				
0–3	1383	43·5	15·9	12·3, 19·7
4–5	978	30·8	15·5	11·8, 19·6
6 or more^*^	817	25·7	14·2	10·5, 18·6
Group 3 – Maternal lifestyle and behaviour factors				
Maternal smoking during pregnancy				
No	3001	94·4	15·2	11·8, 19·3
Yes^*^	177	5·6	15·5	9·6, 21·4
Early-pregnancy binge drinking				
No	3167	99·6	15·2	11·8, 19·5
Yes^*^	11	0·4	17·1	10·5, 20·9
Diet quality – Terciles of the healthy eating index				
1st tercile (32·4–60·2)^*^	1060	33·3	15·0	10·6, 19·5
2nd tercile (60·2–70·6)	1059	33·3	15·4	11·9, 19·5
3rd tercile (> 70·6)	1059	33·3	15·4	12·3, 19·1
Physical activity in the first trimester (MET h/week)				
< 450^*^	1708	53·7	15·0	11·4, 19·5
450–899	610	19·2	15·6	12·2, 20·0
≥ 900	860	27·1	15·3	12·3, 19·1
First-trimester perceived stress				
Low stress (0–13)	1961	61·7	15·5	11·9, 19·3
Moderate stress (14–26)	1123	35·3	15·3	11·4, 19·5
High stress (27–40)^*^	94	3·0	14·3	10·1, 18·7
First-trimester anxiety				
Low anxiety (20–39)	2464	77·6	15·5	11·9, 19·2
Moderate anxiety (40–59)	691	21·7	15·0	11·0, 20·2
High anxiety (60–80)^*^	23	0·7	13·3	7·9, 16·8
First-trimester depressive symptoms				
Not depressed (< 11)	2800	88·1	15·5	11·8, 19·5
Depressed (≥ 11)^*^	378	11·9	14·2	10·5, 19·1
Sleep quality				
Restless^*^	601	18·9	14·6	11·3, 19·5
Average	1300	40·9	15·5	11·7, 19·2
Restful or very restful	1277	40·2	15·5	12·0, 19·5
Group 4 – Adverse neonatal outcomes				
Preterm birth				
No	2930	92·2	15·5	11·9, 19·5
Yes^*^	248	7·8	12·7	9·1, 16·8
Small for gestational age				
No	2921	91·9	15·5	11·9, 19·7
Yes^*^	257	8·1	12·7	9·1, 16·2
Large for gestational age				
No	2985	93·9	15·0	11·8, 19·2
Yes^*^	193	6·1	16·8	13·2, 21·8
Low birth weight				
No	2991	94·1	15·5	11·9, 19·5
Yes^*^	187	5·9	12·0	8·8, 15·5
Macrosomia				
No	2941	92·5	15·0	11·4, 19·1
Yes^*^	237	7·5	17·9	14·1, 21·8
Group 5 – Longer-term postpartum outcomes (2–5 years)				
Excessive postpartum weight retention (kg)				
≤ 5	1876	59·0	14·2	11·0, 17·9
> 5^*^	1302	41·0	17·3	13·1, 21·8
Excessive postpartum weight retention (kg)				
≤ 10	2515	79·1	14·5	11·4, 18·3
> 10^*^	663	20·9	18·6	13·6, 23·6
Child overweight/obesity				
No	2507	78·9	15·1	11·8, 19·1
Yes^*^	671	21·1	15·8	11·6, 20·2
Maternal metabolic syndrome				
No	2662	83·8	15·5	11·9, 19·5
Yes^*^	516	16·2	14·5	9·3, 19·2

GWG, gestational weight gain.

* Refers to the category considered ‘sub-optimal’.

† Weight gain until diagnostic of pre-eclampsia: median: 9·3 (5·9, 13·3) kg; distribution of *z* scores of GWG at delivery: No: 0·05 (–0·54, 0·63)/Yes: 0·43 (–0·13, 0·99).

‡ Weight gain until diagnostic of gestational diabetes: median: 7·7 (3·6, 11·2) kg; distribution of z scores of GWG at delivery: No: 0·07 (–0·50, 0·66)/Yes: −0·15 (–0·75, 0·61).

§ Other diseases included anaemia, hypo/hyperthyroidism, heart diseases, kidney diseases, sickle cell disease, bleeding disorders, autoimmune diseases, mental health conditions, gynaecological conditions and sexually transmitted diseases.

The prevalence of adverse neonatal outcomes was below 10 % for each of the evaluated outcomes, while longer-term adverse outcomes were more common: 41 % of the mothers retained more than 5 kg at 2–5 years, with 21 % retaining more than 10 kg and 16 % had postpartum metabolic syndrome. Twenty-one per cent of the children were classified with overweight or obesity according to BMI.

As expected, weight gain at delivery varied across categories of many of the pre-pregnancy, pregnancy and postpartum factors (Table 2). For example, median weight gain among participants reporting greater nausea/vomiting was 14·2 kg compared with those reporting the least symptoms (15·9 kg), while median weight gain was 16·4 kg among those with an unplanned caesarean delivery compared with 15·0 kg among those without. Nevertheless, the absolute differences in median weight gain between categories were small in magnitude, with differences < 2 kg for most variables. Of note, median weight gain among participants who self-reported a poor diet quality or a sedentary lifestyle was within 0·4 kg of the median weight gain among those who reported a high diet quality or the most active lifestyle. The largest differences in weight gain between categories were observed for gestational diabetes, preterm birth, low birth weight, macrosomia and excess postpartum weight retention.

**Fig. 1.**
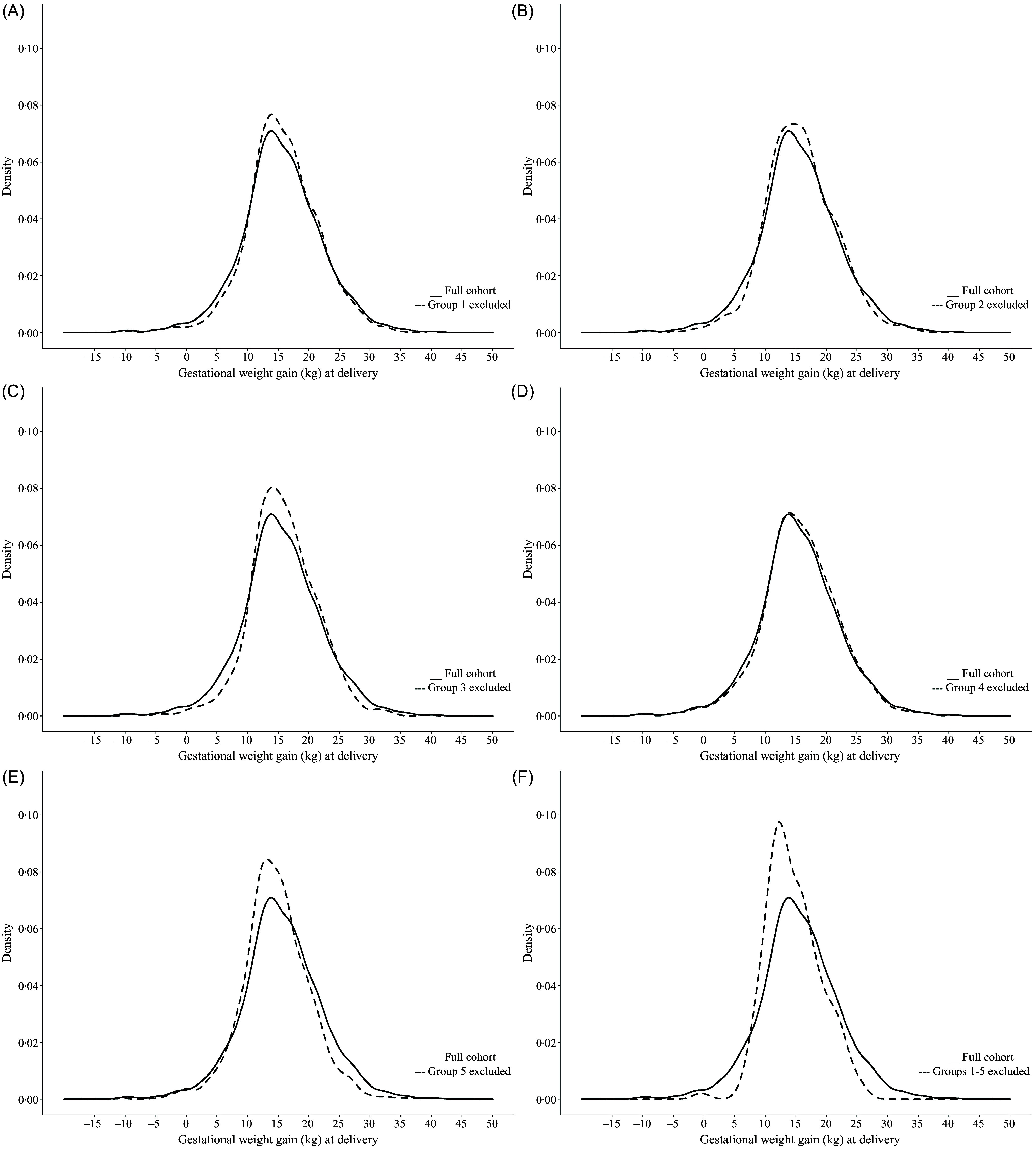
Distribution of gestational weight gain at delivery (26–42 weeks) in the full cohort and after the exclusion of the participants with each of group of conditions. Notes: Group 1: Socio-economic and demographic variables, Group 2: Pre-existing co-morbidities and maternal pregnancy complications, Group 3: Maternal lifestyle and behaviour factors, Group 4: Adverse neonatal outcomes, Group 5: Longer-term postpartum outcomes.

Excluding participants with group 1, 2, 3 or 4 exclusion criteria had virtually no impact on the 25th, median or 75th percentiles of weight gain at delivery (Fig. 1). Weight gain percentiles were only meaningfully different after excluding participants in group 5 (longer-term adverse outcomes). Exclusion of participants in group 5 shifted the weight gain distribution to the left (Fig. 1), with differences most pronounced at the upper end of the distribution: the 75th percentile of weight gain decreased from 19·6 kg in the full cohort to 17·8 kg after participants with excess postpartum weight retention > 5 kg, metabolic syndrome or children with overweight/obesity were excluded. This difference was less pronounced for the weight gain at the second trimester and when excess postpartum weight retention was defined using 10 kg as a cut-off (Table 3). Results are similar when the 95 % CI are considered (online Supplementary Table 2).

**Table 3.** Distribution of gestational weight gain at each study visit in the full and sub-cohorts – all individuals (Median values and interquartile ranges)

Dataset	GWG in the first visit (11–14 weeks)			GWG in the second visit (27–30 weeks)			GWG at delivery (26–42 weeks)		
	Number of measurements^*^	Median	Interquartile range	Number of measurements^*^	Median	Interquartile range	Number of measurements	Median	Interquartile range
Full cohort	2451	2·0	0·4, 3·6	2245	10·0	7·1, 12·9	3178	15·3	11·8, 19·6
Group 1 excluded	1668	2·0	0·6, 3·6	1578	10·0	7·4, 12·7	2181	15·4	12·3, 19·3
Group 2 excluded	734	1·8	0·7, 3·2	669	9·7	7·4, 12·6	936	15·4	11·9, 19·1
Group 3 excluded	709	2·3	0·9, 3·6	639	10·0	8·0, 12·8	909	15·4	12·3, 19·1
Group 4 excluded	1859	2·0	0·4, 3·6	1713	10·0	7·3, 13·1	2430	15·4	12·2, 19·6
Group 5 excluded	1055	1·8	0·4, 3·2	985	9·4	7·1, 11·7	1371	14·5	11·4, 17·8
Group 5b excluded	1371	1·8	0·4, 3·3	1279	9·6	7·3, 12·1	1773	14·9	11·8, 18·6
Groups 1–5 excluded	105	1·8	0·9, 3·0	98	8·8	7·0, 11·4	137	14·0	11·5, 17·0
Groups 1–5b excluded	130	1·8	0·9, 2·9	119	9·1	7·1, 11·4	168	14·1	11·8, 17·3

GWG, gestational weight gain (kg).

Group 1: Socio-economic and demographic variables.

Group 2: Pre-existing co-morbidities and maternal pregnancy complications.

Group 3: Maternal lifestyle and behaviour factors.

Group 4: Adverse neonatal outcomes.

Group 5: Longer-term postpartum outcomes (weight retention > 5 kg).

Group 5b: Longer-term postpartum outcomes (weight retention > 10 kg).

* Sample sizes for visits 1 and 2 vary due to the selection of weeks, missing data and outliers of GWG in each visit.

Examination of the individual exclusion criteria within group 5 (longer-term adverse outcomes) suggested that the exclusion of participants with excess postpartum weight retention was responsible for influencing weight gain percentiles. For example, the 75th percentile in the full cohort was 19·6, decreasing to 17·9 and 18·3 kg after excluding participants with excess postpartum weight retention > 5 kg > 10 kg, respectively. Exclusion of children with overweight/obesity or participants with metabolic syndrome did not meaningfully alter the weight gain distribution (Table 4).

**Table 4.** Distribution of gestational weight gain at delivery according to the variables of the groups with meaningful differences (group 5 – longer-term postpartum outcomes) (Median values and interquartile ranges)

Dataset	GWG in the first visit (11–14 weeks weeks)			GWG in the second visit (27–30 weeks)			GWG at delivery (26–42 weeks)		
	Number of measurements	Median	Interquartile range	Number of measurements	Median	Interquartile range	Number of measurements	Median	Interquartile range
Full cohort	2451	2·0	0·4, 3·6	2245	10·0	7·1, 12·9	3178	15·3	11·8, 19·6
Weight retention > 5 kg excluded	1449	1·8	0·3, 3·2	1343	9·2	6·8, 11·8	1876	14·2	11·0, 17·9
Weight retention > 10 kg excluded	1942	1·8	0·4, 3·3	1797	9·5	6·9, 12·1	2515	14·5	11·4, 18·3
Child overweight/obesity excluded	1940	2·0	0·4, 3·6	1769	10·0	7·2, 12·7	2507	15·1	11·8, 19·1
Maternal metabolic syndrome excluded	2065	2·0	0·4, 3·6	1890	10·0	7·3, 12·7	2662	15·4	11·9, 19·5
Group 5 excluded	1055	1·8	0·4, 3·2	985	9·4	7·1, 11·7	1371	14·5	11·4, 17·8
Group 5b excluded	1371	1·8	0·4, 3·3	1279	9·6	7·3, 12·1	1773	14·9	11·8, 18·6

GWG, gestational weight gain (kg).

The exclusion of individuals with any of the pre-pregnancy, pregnancy or postpartum risk factors potentially related to sub-optimal weight gain (i.e. in any of groups 1–5) left only 137 participants (approximately 4 % of the original cohort). The median and the 75th percentile for weight gain at delivery between the full cohort and the sub-cohort with all exclusion criteria applied differed by > 0·75 kg (Table 3), and there was a shift to lower values in the weight gain distribution (Fig. 1(f)). These changes were due to the exclusion of participants in group 5 (longer-term adverse outcomes). Similar results were observed for the *z* scores of weight gain at delivery (online Supplementary Table 3).

When analyses were stratified according to pre-pregnancy BMI, similar results were also observed for participants with normal weight (online Supplementary Fig. 3 and online Supplementary Table 4). For participants with overweight and obesity, the exclusion of participants with group 3 characteristics (maternal lifestyle and behaviour) caused a shift in the distribution of weight gain at delivery (online Supplementary Figures 4 and 5). However, these differences may be chance findings given the lower sample size in these two BMI categories (online Supplementary Table 4). Only group 5 exclusion criteria (longer-term adverse outcomes), and more specifically, excess postpartum weight retention (> 5 and > 10 kg), changed the weight gain distribution in all BMI categories (online Supplementary Tables 5–7).

## Discussion

In this large US cohort of nulliparous pregnancies, we found that excess postpartum weight retention (> 5 or > 10 kg) was the only exclusion criterion to meaningfully impact weight gain percentiles. Exclusion of participants with excess postpartum weight retention lowered 75th percentile values by approximately 2 kg. Otherwise, pregnancy weight gain percentiles were not meaningfully impacted by the exclusion of participants with adverse pre-pregnancy, pregnancy or postpartum factors linked with sub-optimal weight gain. This suggests that most potential exclusion criteria for a pregnancy weight gain standard may have little practical impact on the chart percentiles, although our analysis cannot rule out an impact on the extremes of the distribution.

Our finding that most exclusion criteria have little practical impact on the percentile values of a pregnancy weight gain chart was unexpected, given that the selected factors are recognised in the literature as determinants or outcomes of weight gain. Yet, similar findings have been observed in the context of fetal weight charts^(23)^. Hutcheon and Liauw observed that the distribution of estimated foetal weight at 32–33 weeks’ gestation, including the value of the 10th percentile used to define small-for-gestational-age birth, was virtually identical in a reference population and a standard population to which an extensive list of exclusion criteria had been applied^(38)^.

Our team has recently evaluated the impact of excluding pregnancies with adverse neonatal outcomes on the resulting weight gain percentiles using studies conducted in low- and middle-income countries^(39)^. We found that chart percentiles for normal and overweight pregnant women and individuals was unchanged when those who gave birth to neonates with adverse outcomes were removed from the sample^(39)^. Our team also evaluated the impact of including women and individuals with high interpregnancy weight change (as a proxy for excess postpartum weight retention) on the percentile values of GWG charts in a large, population-based cohort from Sweden^(40)^. In contrast with our findings in this cohort, exclusion of pregnant women and other individuals with high interpregnancy weight change had no impact on chart percentiles. The prevalence of interpregnancy weight change ≥ 5 kg in the Swedish data was lower than we observed in this study (34 *v*. 40 %), potentially explaining the discrepancy in findings.

We speculate that the extent to which a potential exclusion criterion influences the values of percentiles depends on both its prevalence and strength of association with GWG. Although several of the groups of variables in our study had high combined prevalences (e.g. groups 2 and 3 excluded > 70 % of the cohort), they did not meaningfully impact the distribution of weight gain throughout pregnancy due to their low strength of the association with weight gain (as seen in Table 2). The sole exclusion criterion to meaningfully impact weight gain percentiles, excess postpartum weight retention, was both strongly associated with weight gain and had a high prevalence among the participants of the current study.

Another important finding of this study was that only 4 % of the original cohort remained after applying all possible exclusion criteria. This decrease in sample size highlights the practical burden that extensive exclusion criteria create at the data collection stage when using a prospective cohort for chart creation. It also raises concerns about the generalisability of the cohort that remains after applying extensive exclusion criteria. Although pregnancy weight gain standards are not intended to be constructed with representative samples, a chart created using only 4 % of the original population may raise concerns with face validity.

Existing pregnancy weight gain charts^(8–16)^ have varied in their inclusion/exclusion criteria. However, none of the existing pregnancy weight gain charts, including the standards proposed by the INTERGROWTH-21st project^(11)^, excluded participants with excess postpartum weight retention. Our findings suggest that published charts may therefore normalise higher weight gains that may in practice increase an individual’s risk of excess postpartum weight retention.

The primary strength of this study is the availability of detailed information on maternal psychosocial factors, diet quality, physical activity and longer-term maternal and child health outcomes. These variables allowed us to explore the impact of a detailed list of possible exclusion criteria on weight gain percentiles. Nevertheless, our sample size was not large enough to evaluate the impact of those variables in underweight individuals, and the final sample size for women and individuals with overweight and obesity was also low (< 20). Further, the NuMoM2b/HHS only included nulliparous women, potentially limiting generalisability if the association between possible exclusion criteria and weight gain differs by parity, or the prevalence of the exclusion criteria is meaningfully different in parous women. Finally, regarding diet and physical activity, although they were obtained based on self-reported information and are prone to error, those types of instruments are likely what would be used in a real-world prospective study seeking to create a standard, due to practical challenges in obtaining more objective measures in large population cohorts.

Most potential inclusion/exclusion criteria for a pregnancy weight gain standard will have little practical impact on the resulting chart percentiles. However, pregnant women and other individuals with excess postpartum weight retention should be excluded from cohorts used to create pregnancy weight gain standards, as retaining these individuals will result in chart percentiles that are up to 2 kg higher. Future simulation studies may be useful in identifying the combination of a characteristic’s prevalence and its strength of association with pregnancy weight gain necessary for an exclusion criterion to influence the percentile values of pregnancy weight gain standards. Repeating this analysis in studies from different global settings and integrating findings is a fundamental step towards informing the inclusion/exclusion criteria for global weight gain standards.

## Supporting information

Rangel Bousquet Carrilho et al. supplementary material Rangel Bousquet Carrilho et al. supplementary material

## Supplementary material

For supplementary material/s referred to in this article, please visit https://doi.org/10.1017/S0007114524001855
