# Supplementary material for: The impact of cohort inclusion/exclusion criteria on pregnancy weight gain chart percentiles
Source: Br J Nutr. 2024 Oct 2;132(6):751–61. doi: 10.1017/S0007114524001855 (PMC11557291; doi:10.1017/S0007114524001855)
Supplement: Rangel Bousquet Carrilho et al. supplementary material [file S0007114524001855sup001.docx]

**The impact of cohort inclusion/exclusion criteria on pregnancy weight gain chart percentiles**

**SUPPLEMENTARY MATERIAL**

**Table of contents**

[**Supplementary Figure 1.** Flowchart for the construction of the final dataset used in this study. 2](#_Toc169084875)

[**Supplementary Figure 2.** Distribution of gestational weight gain (without missing weight gain data) in the complete HHS dataset vs. full cohort vs. dataset with individuals with missing data in key variables. 3](#_Toc169084876)

[**Supplementary Table 1.** Percentage of missing data in the key selected variables according to the groups. 4](#_Toc169084877)

[**Supplementary Table 2.** Point estimates and 95% confidence intervals for the median and quantiles for groups with meaningful differences (> 1 kg) in gestational weight gain distribution. 5](#_Toc169084878)

[**Supplementary Table 3.** Distribution of gestational weight gain in *z* scores at delivery in the full and sub-cohorts – all individuals. 6](#_Toc169084879)

[**Supplementary Table 4.** Distribution of gestational weight gain at each study visit in the full and sub-cohorts according to pre-pregnancy BMI categories (normal weight, overweight, and obesity). 7](#_Toc169084880)

[**Supplementary Table 5.** Distribution of gestational weight gain at delivery according to the variables of the groups with meaningful differences (Group 5 – Longer-term postpartum outcomes) for individuals with pre-pregnancy normal weight. 9](#_Toc169084881)

[**Supplementary Table 6.** Distribution of gestational weight gain at delivery according to the variables of the groups with meaningful differences (Groups 2, 3, 5 and 5b) for individuals with pre-pregnancy overweight. 10](#_Toc169084882)

[**Supplementary Table 7.** Distribution of gestational weight gain at delivery according to the variables of the groups with meaningful differences (Groups 1, 2, 3, 5 and 5b) for individuals with pre-pregnancy obesity. 12](#_Toc169084883)

[**Supplementary Figure 3.** Distribution of gestational weight gain at delivery (26 – 42 weeks) in the full cohort and after the exclusion of the participants with each of group of conditions – individuals with pre-pregnancy normal weight. 15](#_Toc169084884)

[**Supplementary Figure 4.** Distribution of gestational weight gain at delivery (26 – 42 weeks) in the full cohort and after the exclusion of the participants with each of group of conditions – individuals with pre-pregnancy overweight. 16](#_Toc169084885)

[**Supplementary Figure 5.** Distribution of gestational weight gain at delivery (26 – 42 weeks) in the full cohort and after the exclusion of the participants with each of group of conditions – individuals with pre-pregnancy obesity. 17](#_Toc169084886)


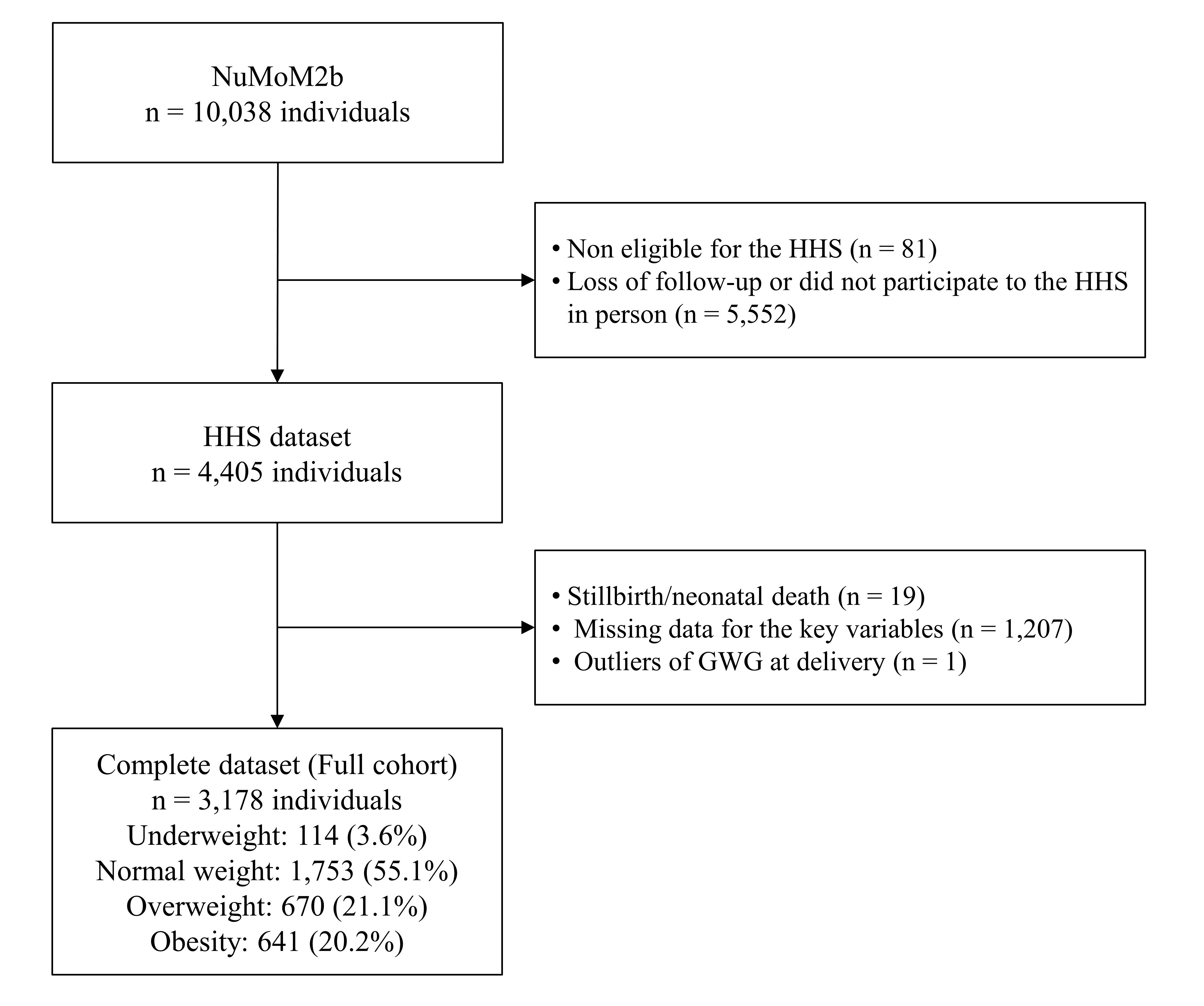


# **Supplementary Figure 1.** Flowchart for the construction of the final dataset used in this study.

Note: HHS: Heart health study; GWG: gestational weight gain.


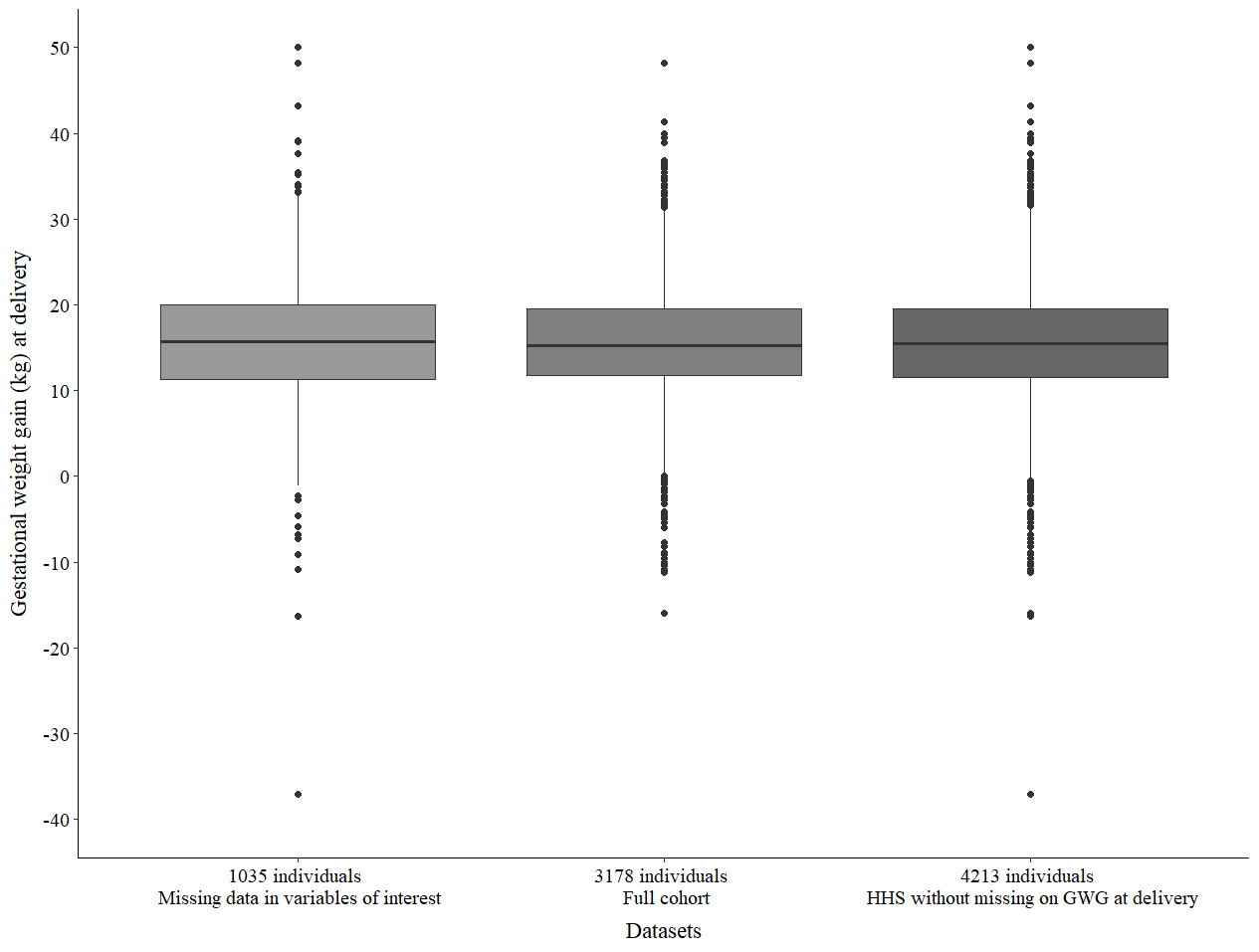


# **Supplementary Figure 2.** Distribution of gestational weight gain (without missing weight gain data) in the complete HHS dataset vs. full cohort vs. dataset with individuals with missing data in key variables.

Note: Sample sizes were obtained as follows:

a. n = 1035: individuals with missing data in one or more variables of the selected groups

b. n = 3178: full cohort – individuals without missing data in key variables and in GWG at delivery

c. n = 4213: complete HHS data (4405 individuals from the HHS - 19 stillbirth/neonatal death - 169 missing GWG at delivery - 4 outliers of GWG at delivery)

# **Supplementary Table 1.** Percentage of missing data in the key selected variables according to the groups.

| **Selected variables** | **n (%)** |
| --- | --- |
| Pre-pregnancy BMI | 7 (0.2) |
| GWG at delivery | 169 (3.8) |
| Gestational age at delivery | 0 |
|  |  |
| *Group 1 – Socioeconomic and demographic variables* | |
| Maternal age | 0 |
| Maternal education | 0 |
| Public health insurance | 3 (0.1) |
|  |  |
| *Group 2 – Pre-existing comorbidities and maternal pregnancy complications* | |
| Pre-pregnancy hypertension | 0 |
| Pre-eclampsia | 7 (0.2) |
| Pre-pregnancy diabetes | 0 |
| Gestational diabetes | 0 |
| Other diseases | 5 (0.1) |
| Unplanned cesarean section | 3 (0.1) |
| Nausea and vomiting (PUQE score) | 1 (0.02) |
|  |  |
| *Group 3 – Maternal lifestyle and behaviour factors* | |
| Maternal smoking during pregnancy | 32 (0.7) |
| Early-pregnancy binge drinking | 123 (2.8) |
| Diet quality (terciles of the healthy eating index) | 641 (14.6) |
| Physical activity in the first trimester | 10 (0.2) |
| First-trimester perceived stress | 18 (0.4) |
| First-trimester maternal anxiety | 452 (10.3) |
| First-trimester depressive symptoms | 106 (2.4) |
| Sleep quality | 480 (10.9) |
|  |  |
| *Group 4 – Adverse neonatal outcomes* | |
| Preterm birth | 0 |
| Small for gestational age | 13 (0.3) |
| Large for gestational age | 13 (0.3) |
| Birth weight | 13 (0.3) |
|  |  |
| *Group 5 – Longer-term postpartum outcomes* | |
| Postpartum weight retention | 16 (0.4) |
| Child overweight/obesity | 144 (3.3) |
| Maternal metabolic syndrome | 0 |

Notes: BMI: Body mass index; GWG: gestational weight gain. Percentage was calculated based on n = 4386 (4405 from the HHS – 19 stillbirth/neonatal death).

# **Supplementary Table 2.** Point estimates and 95% confidence intervals for the median and quantiles for groups with meaningful differences (> 0.75 kg) in gestational weight gain distribution.

| Dataset | GWG in the second visit  (27 – 30 weeks) | | | GWG at delivery  (26 – 42 weeks) | | |
| --- | --- | --- | --- | --- | --- | --- |
|  | P25 (95% CI) | Median (95% CI) | P75 (95% CI) | P25 (95% CI) | Median (95% CI) | P75 (95% CI) |
| Full cohort | 7.1 (6.9 – 7.4) | 10.0 (9.7 – 10.2) | 12.9 (12.6 – 13.2) | 11.8 (11.6 – 12.0) | 15.3 (15.0 – 15.5) | 19.6 (19.2 – 19.8) |
| Group 5 excluded | 7.1 (6.8 – 7.4) | 9.4 (9.1 – 9.7) | 11.7 (11.3 – 12.0) | 11.4 (11.0 – 11.7) | 14.5 (14.2 – 14.9) | 17.8 (17.4 – 18.3) |
| Group 5b excluded | 7.3 (7.0 – 7.5) | 9.6 (9.4 – 9.9) | 12.1 (11.8 – 12.4) | 11.8 (11.5 – 12.1) | 14.9 (14.6 – 15.2) | 18.6 (18.3 – 19.0) |
| Groups 1-5 excluded | 7.0 (6.2 – 7.7) | 8.8 (8.1 – 9.6) | 11.4 (10.2 – 12.5) | 11.5 (10.8 – 12.2) | 14.0 (13.1 – 15.0) | 17.0 (15.6 – 18.4) |
| Groups 1-5b excluded | 7.1 (6.3 – 7.7) | 9.1 (8.3 – 9.8) | 11.4 (10.4 – 12.5) | 11.8 (11.1 – 12.5) | 14.1 (13.2 – 15.0) | 17.3 (16.2 – 18.4) |

Notes: CI: confidence interval; GWG: Gestational weight gain (kg).

Group 1: Socioeconomic and demographic variables

Group 2: Pre-existing comorbidities and maternal pregnancy complications

Group 3: Maternal lifestyle and behaviour factors

Group 4: Adverse neonatal outcomes

Group 5: Longer-term postpartum outcomes (weight retention > 5kg)

Group 5b: Longer-term postpartum outcomes (weight retention > 10 kg)

# **Supplementary Table 3.** Distribution of gestational weight gain in *z* scores at delivery in the full and sub-cohorts – all individuals.

| **Dataset** | ***z* scores of GWG at delivery** | |
| --- | --- | --- |
|  | **Number of measurements** | **Median**  **[interquartile range]** |
| Full cohort | 3178 | 0.07 [-0.51; 0.66] |
| Group 1 excluded | 2181 | 0.07 [-0.45; 0.62] |
| Group 2 excluded | 936 | 0.03 [-0.57; 0.60] |
| Group 3 excluded | 909 | 0.02 [-0.51; 0.60] |
| Group 4 excluded | 2430 | 0.07 [-0.50; 0.66] |
| Group 5 excluded | 1371 | -0.16 [-0.70; 0.42] |
| Group 5b excluded | 1773 | -0.07 [-0.64; 0.50] |
| Groups 1-5 excluded | 137 | -0.32 [-0.79; 0.27] |
| Groups 1-5b excluded | 168 | -0.20 [-0.75; 0.27] |

Notes: GWG: Gestational weight gain (kg).

Group 1: Socioeconomic and demographic variables

Group 2: Pre-existing comorbidities and maternal pregnancy complications

Group 3: Maternal lifestyle and behaviour factors

Group 4: Adverse neonatal outcomes

Group 5: Longer-term postpartum outcomes (weight retention > 5kg)

Group 5b: Longer-term postpartum outcomes (weight retention > 10 kg)

# **Supplementary Table 4.** Distribution of gestational weight gain at each study visit in the full and sub-cohorts according to pre-pregnancy BMI categories (normal weight, overweight, and obesity).

| Dataset | GWG in the first visit  (11 – 14 weeks) | | GWG in the second visit  (27 – 30 weeks) | | GWG at delivery  (26 – 42 weeks) | |
| --- | --- | --- | --- | --- | --- | --- |
|  | Number of measurements* | Median [interquartile range] | Number of measurements* | Median [interquartile range] | Number of measurements | Median [interquartile range] |
| *Normal weight* | | | | | | |
| Full cohort | 1355 | 2.1 [0.7, 3.5] | 1249 | 10.4 [8.1, 12.9] | 1753 | 15.9 [12.6, 19.5] |
| Group 1 excluded | 1005 | 2.0 [0.9, 3.3] | 952 | 10.3 [8.1, 12.7] | 1315 | 15.9 [12.7, 19.1] |
| Group 2 excluded | 482 | 1.9 [0.8, 3.2] | 436 | 9.7 [7.7, 12.6] | 609 | 15.6 [12.3, 19.1] |
| Group 3 excluded | 477 | 2.3 [0.9, 3.4] | 428 | 10.0 [8.3, 12.4] | 611 | 15.4 [12.7, 18.8] |
| Group 4 excluded | 1072 | 2.1 [0.7, 3.5] | 983 | 10.4 [8.2, 13.2] | 1387 | 15.9 [12.7, 19.6] |
| Group 5 excluded | 734 | 1.8 [0.5, 3.0] | 696 | 9.7 [7.7, 11.9] | 955 | 15.0 [11.9, 18.2] |
| Group 5b excluded | 934 | 2.0 [0.7, 3.2] | 887 | 10.0 [7.7, 12.5] | 1210 | 15.4 [12.3, 18.7] |
| Groups 1-5 excluded | 83 | 2.0 [0.9, 3.0] | 78 | 9.0 [7.4, 11.4] | 106 | 14.1 [11.7, 17.2] |
| Groups 1-5b excluded | 99 | 2.0 [0.9, 3.0] | 92 | 9.1 [7.3, 11.4] | 127 | 14.1 [11.8, 17.3] |
|  |  |  |  |  |  |  |
| *Overweight* | | | | | | |
| Full cohort | 512 | 2.3 [0.4, 4.4] | 465 | 10.4 [7.3, 13.9] | 670 | 15.6 [12.3, 20.7] |
| Group 1 excluded | 326 | 2.3 [0.6, 4.3] | 308 | 10.8 [7.7, 14.3] | 437 | 15.9 [12.7, 20.9] |
| Group 2 excluded | 132 | 1.8 [0.4, 3.3] | 128 | 9.9 [7.5, 12.7] | 180 | 15.4 [12.4, 20.1] |
| Group 3 excluded | 136 | 2.4 [0.9, 4.5] | 116 | 10.9 [8.1, 14.5] | 166 | 16.4 [13.6, 21.8] |
| Group 4 excluded | 376 | 2.2 [0.4, 4.3] | 353 | 10.4 [7.5, 13.7] | 509 | 15.9 [12.4, 20.7] |
| Group 5 excluded | 167 | 1.4 [0, 2.9] | 147 | 8.3 [5.9, 11.0] | 219 | 13.7 [11.3, 16.8] |
| Group 5b excluded | 232 | 1.8 [0.3, 3.3] | 213 | 9.0 [6.9, 11.6] | 303 | 14.5 [11.4, 18.2] |
| Groups 1-5 excluded | 12 | 0.9 [0.1, 1.6] | 11 | 8.6 [7.0, 11.4] | 17 | 15.0 [12.5, 16.9] |
| Groups 1-5b excluded | 15 | 0.9 [-0.1, 2.5] | 14 | 8.4 [7.8, 11.7] | 21 | 15.0 [13.6, 16.9] |
|  |  |  |  |  |  |  |
| *Obesity* | | | | | | |
| Full cohort | 497 | 1.4 [-1.1, 3.7] | 458 | 7.6 [3.6, 11.8] | 641 | 12.8 [7.5, 18.2] |
| Group 1 excluded | 288 | 1.8 [-0.4, 3.6] | 275 | 7.7 [4.5, 11.8] | 364 | 12.8 [8.5, 17.7] |
| Group 2 excluded | 89 | 2.0 [0.2, 3.8] | 83 | 8.6 [5.0, 12.4] | 111 | 13.6 [9.5, 18.4] |
| Group 3 excluded | 74 | 1.4 [-0.3, 3.5] | 79 | 8.6 [5.0, 13.0] | 105 | 14.5 [7.7, 18.6] |
| Group 4 excluded | 344 | 1.1 [-1.4, 3.4] | 318 | 7.8 [3.6, 12.0] | 443 | 13.2 [7.7, 18.0] |
| Group 5 excluded | 96 | 0.9 [-1.7, 2.7] | 95 | 6.4 [2.7, 10.0] | 126 | 12.3 [6.8, 16.4] |
| Group 5b excluded | 137 | 0.9 [-1.4, 2.7] | 124 | 6.7 [3.1, 10.0] | 174 | 12.3 [7.7, 16.8] |
| Groups 1-5 excluded | 4 | -0.6 [-2.5, 1.6] | 6 | 7.0 [5.0, 9.2] | 6 | 12.4 [10.2, 15.4] |
| Groups 1-5b excluded | 8 | 1.5 [0.1, 2.8] | 9 | 7.7 [4.5, 10.0] | 10 | 12.4 [9.9, 17.6] |

Notes: GWG: Gestational weight gain (kg).

*Sample sizes for visits 1 and 2 vary due to the selection of weeks, missing data, and outliers of GWG in each visit.

Median (IQR) BMI for each pre-pregnancy BMI category: Normal weight = 22.1 [20.7, 23.3]; Overweight = 27.0 [25.9, 28.3]; Obesity: 34.8 [31.9, 38.8].

Group 1: Socioeconomic and demographic variables

Group 2: Pre-existing comorbidities and maternal pregnancy complications

Group 3: Maternal lifestyle and behaviour factors

Group 4: Adverse neonatal outcomes

Group 5: Longer-term postpartum outcomes (weight retention > 5kg)

Group 5b: Longer-term postpartum outcomes (weight retention > 10 kg)

# **Supplementary Table 5.** Distribution of gestational weight gain at delivery according to the variables of the groups with meaningful differences (Group 5 – Longer-term postpartum outcomes) for individuals with pre-pregnancy normal weight.

| Dataset | GWG in the second visit  (27 – 30 weeks) | | GWG at delivery  (26 – 42 weeks) | |
| --- | --- | --- | --- | --- |
|  | Number of measurements | Median  [interquartile range] | Number of measurements | Median  [interquartile range] |
| Full cohort | 1249 | 10.4 [8.1, 12.9] | 1753 | 15.9 [12.6, 19.5] |
| Weight retention > 5 kg excluded | 848 | 9.8 [7.7, 11.9] | 1169 | 15.0 [12.2, 18.4] |
| Weight retention > 10 kg excluded | 1097 | 10.0 [7.9, 12.6] | 1516 | 15.4 [12.3, 18.8] |
| Child overweight/obesity excluded | 1051 | 10.4 [7.9, 12.9] | 1462 | 15.9 [12.3, 19.4] |
| Maternal metabolic syndrome excluded | 1165 | 10.4 [8.1, 12.7] | 1628 | 15.9 [12.5, 19.5] |
| Group 5 excluded | 696 | 9.7 [7.7, 11.9] | 955 | 15.0 [11.9, 18.2] |
| Group 5b excluded | 887 | 10.0 [7.7, 12.5] | 1210 | 15.4 [12.3, 18.7] |

Note: GWG: Gestational weight gain (kg).

# **Supplementary Table 6.** Distribution of gestational weight gain at delivery according to the variables of the groups with meaningful differences (Groups 2, 3, 5 and 5b) for individuals with pre-pregnancy overweight.

| Dataset | GWG in the first visit  (11 – 14 weeks weeks) | | GWG in the second visit  (27 – 30 weeks) | | GWG at delivery  (26 – 42 weeks) | |
| --- | --- | --- | --- | --- | --- | --- |
|  | Number of measurements* | Median [interquartile range] | Number of measurements* | Median [interquartile range] | Number of measurements | Median [interquartile range] |
| Full cohort | 512 | 2.3 [0.4, 4.4] | 465 | 10.4 [7.3, 13.9] | 670 | 15.6 [12.3, 20.7] |
|  |  |  |  |  |  |  |
| *Group 2 – Pre-existing comorbidities and maternal pregnancy complications* | | | | | | |
| Pre-pregnancy hypertension excluded | 493 | 2.3 [0.4, 4.5] | 451 | 10.4 [7.3, 14.1] | 648 | 15.8 [12.3, 20.9] |
| Pre-eclampsia excluded | 470 | 2.3 [0.4, 4.3] | 431 | 10.3 [7.3, 13.8] | 617 | 15.4 [12.3, 20.5] |
| Pre-pregnancy diabetes excluded | 506 | 2.3 [0.4, 4.5] | 460 | 10.4 [7.3, 14.0] | 663 | 15.6 [12.3, 20.9] |
| Gestational diabetes excluded | 481 | 2.3 [0.4, 4.4] | 441 | 10.1 [7.3, 13.9] | 633 | 15.9 [12.3, 20.6] |
| Other diseases excluded | 277 | 2.0 [0.4, 4.3] | 256 | 10.1 [7.5, 13.5] | 368 | 15.5 [11.8, 20.5] |
| Unplanned caesarean section excluded | 388 | 1.9 [0.3, 4.1] | 353 | 9.9 [7.0, 13.2] | 517 | 15.0 [11.8, 20.0] |
| Severe nausea and vomiting excluded | 386 | 2.3 [0.9, 4.3] | 355 | 10.4 [7.7, 14.3] | 491 | 16.3 [12.5, 20.9] |
| Group 2 excluded | 132 | 1.8 [0.4, 3.3] | 128 | 9.9 [7.5, 12.7] | 180 | 15.4 [12.4, 20.1] |
|  |  |  |  |  |  |  |
| *Group 3 – Maternal lifestyle and behaviour factors* | | | | | | |
| Smoking during pregnancy excluded | 485 | 2.3 [0.4, 4.4] | 437 | 10.4 [7.4, 13.9] | 634 | 15.8 [12.3, 20.6] |
| Early-pregnancy binge drinking excluded | 510 | 2.3 [0.4, 4.4] | 463 | 10.4 [7.3, 14.0] | 665 | 15.6 [12.3, 20.7] |
| Poor diet quality excluded | 340 | 2.6 [0.9, 4.5] | 309 | 10.9 [7.9, 14.4] | 446 | 15.9 [12.7, 20.9] |
| Physical inactivity during pregnancy excluded | 222 | 2.6 [0.6, 4.4] | 194 | 10.9 [7.5, 14.7] | 293 | 15.9 [12.7, 21.4] |
| Maternal stress excluded | 489 | 2.3 [0.5, 4.4] | 443 | 10.4 [7.4, 14.1] | 639 | 15.9 [12.3, 20.9] |
| Maternal anxiety excluded | 509 | 2.3 [0.4, 4.4] | 460 | 10.4 [7.3, 14.0] | 665 | 15.6 [12.3, 20.7] |
| Maternal depressive symptoms excluded | 437 | 2.3 [0.5, 4.5] | 403 | 10.4 [7.7, 14.1] | 573 | 16.0 [12.5, 20.7] |
| Poor sleep quality excluded | 408 | 2.2 [0.4, 4.5] | 370 | 10.4 [7.4, 14.0] | 521 | 15.7 [12.3, 20.9] |
| Group 3 excluded | 136 | 2.4 [0.9, 4.5] | 116 | 10.9 [8.1, 14.5] | 166 | 16.4 [13.6, 21.8] |
|  |  |  |  |  |  |  |
| *Group 5 – Longer-term postpartum outcomes* | | | | | | |
| Weight retention > 5 kg excluded | 255 | 1.6 [-0.3, 3.2] | 224 | 8.3 [5.9, 11.6] | 334 | 13.8 [10.9, 16.9] |
| Child overweight and obesity excluded | 388 | 2.2 [0.5, 4.1] | 350 | 10.0 [7.4, 13.6] | 506 | 15.2 [12.3, 20.4] |
| Maternal metabolic syndrome excluded | 406 | 2.2 [0.5, 4.3] | 369 | 10.1 [7.4, 13.6] | 533 | 15.5 [12.3, 20.4] |
| Group 5 excluded | 167 | 1.4 [0, 2.9] | 147 | 8.3 [5.9, 11.0] | 219 | 13.7 [11.3, 16.8] |
|  |  |  |  |  |  |  |
| *Group 5 – Longer-term postpartum outcomes* | | | | | | |
| Weight retention > 10 kg excluded | 360 | 1.8 [0.1, 3.4] | 327 | 9.1 [6.4, 12.1] | 473 | 14.3 [11.4, 18.2] |
| Child overweight and obesity excluded | 388 | 2.2 [0.5, 4.1] | 350 | 10.0 [7.4, 13.6] | 506 | 15.2 [12.3, 20.4] |
| Maternal metabolic syndrome excluded | 406 | 2.2 [0.5, 4.3] | 369 | 10.1 [7.4, 13.6] | 533 | 15.5 [12.3, 20.4] |
| Group 5b excluded | 232 | 1.8 [0.3, 3.3] | 213 | 9.0 [6.9, 11.6] | 303 | 14.5 [11.4, 18.2] |

Notes: GWG: Gestational weight gain (kg). *Sample sizes for visits 1 and 2 vary due to the selection of weeks, missing data, and outliers of GWG in each visit.

# **Supplementary Table 7.** Distribution of gestational weight gain at delivery according to the variables of the groups with meaningful differences (Groups 1, 2, 3, 5 and 5b) for individuals with pre-pregnancy obesity.

| Dataset | GWG in the first visit  (11 – 14 weeks weeks) | | GWG in the second visit  (27 – 30 weeks) | | GWG at delivery  (26 – 42 weeks) | |
| --- | --- | --- | --- | --- | --- | --- |
|  | Number of measurements* | Median [interquartile range] | Number of measurements* | Median [interquartile range] | Number of measurements | Median [interquartile range] |
| Full cohort | 497 | 1.4 [-1.1, 3.7] | 458 | 7.6 [3.6, 11.8] | 641 | 12.8 [7.5, 18.2] |
|  |  |  |  |  |  |  |
| *Group 1 – Socioeconomic and demographic variables* | | | | | | |
| Maternal age ≤ 19.0 and > 39 y old excluded | 446 | 1.4 [0.9, 3.7] | 408 | 7.6 [3.7, 11.8] | 574 | 12.8 [7.3, 17.7] |
| Maternal education ≤ High School Grad excluded | 393 | 1.7 [-0.7, 3.7] | 365 | 7.7 [3.7, 11.7] | 503 | 12.7 [7.9, 17.7] |
| Public/self-pay health insurance excluded | 321 | 1.8 [-0.6, 3.6] | 312 | 7.7 [4.1, 11.9] | 416 | 12.9 [8.2, 17.7] |
| Group 1 excluded | 288 | 1.8 [-0.4, 3.6] | 275 | 7.7 [4.5, 11.8] | 364 | 12.8 [8.5, 17.7] |
|  |  |  |  |  |  |  |
| *Group 2 – Pre-existing comorbidities and maternal pregnancy complications* | | | | | | |
| Pre-pregnancy hypertension excluded | 434 | 1.3 [-1.3, 3.7] | 407 | 7.6 [3.6, 11.9] | 566 | 12.8 [7.7, 18.2] |
| Pre-eclampsia excluded | 415 | 1.3 [-1.3, 3.6] | 391 | 7.6 [3.6, 11.7] | 535 | 12.6 [7.3, 17.7] |
| Pre-pregnancy diabetes excluded | 479 | 1.4 [-1.2, 3.6] | 444 | 7.6 [3.6, 11.7] | 617 | 12.7 [7.3, 18.0] |
| Gestational diabetes excluded | 454 | 1.4 [-1.3, 3.6] | 413 | 7.4 [3.6, 11.7] | 581 | 13.2 [7.7, 18.2] |
| Other diseases excluded | 236 | 1.5 [-1.4, 3.8] | 214 | 7.6 [3.2, 11.9] | 309 | 12.6 [7.3, 18.0] |
| Unplanned caesarean section excluded | 333 | 1.3 [-1.0, 3.6] | 301 | 7.3 [3.6, 12.0] | 430 | 12.7 [7.3, 18.2] |
| Severe nausea and vomiting excluded | 360 | 1.9 [-0.4, 4.1] | 330 | 7.3 [3.6, 12.0] | 449 | 13.6 [8.4, 18.5] |
| Group 2 excluded | 89 | 2.0 [0.2, 3.8] | 83 | 8.6 [5.0, 12.4] | 111 | 13.6 [9.5, 18.4] |
|  |  |  |  |  |  |  |
| *Group 3 – Maternal lifestyle and behaviour factors* | | | | | | |
| Smoking during pregnancy excluded | 456 | 1.4 [-1.1, 3.7] | 420 | 7.7 [3.7, 11.7] | 583 | 12.7 [7.7, 17.7] |
| Early-pregnancy binge drinking excluded | 496 | 1.4 [-1.2, 3.7] | 457 | 7.4 [3.4, 11.8] | 638 | 12.8 [7.6, 18.1] |
| Poor diet quality excluded | 292 | 1.6 [-0.9, 4.0] | 271 | 8.4 [5.0, 12.1] | 369 | 13.6 [8.6, 17.9] |
| Physical inactivity during pregnancy excluded | 171 | 0.9 [-1.2, 3.2] | 468 | 7.9 [3.6, 12.1] | 229 | 13.6 [8.2, 18.7] |
| Maternal stress excluded | 479 | 1.4 [-1.1, 3.7] | 445 | 7.4 [3.4, 11.7] | 615 | 12.7 [7.6, 17.8] |
| Maternal anxiety excluded | 494 | 1.4 [-1.2, 3.7] | 455 | 7.4 [3.5, 11.8] | 634 | 12.8 [7.7, 18.2] |
| Maternal depressive symptoms excluded | 422 | 1.4 [-1.2, 3.8] | 397 | 7.6 [3.6, 11.8] | 549 | 13.0 [7.7, 17.9] |
| Poor sleep quality excluded | 374 | 1.7 [-0.9, 4.1] | 348 | 8.2 [4.1, 12.3] | 487 | 13.6 [7.8, 18.6] |
| Group 3 excluded | 74 | 1.4 [-0.3, 3.5] | 79 | 8.6 [5.0, 13.0] | 105 | 14.5 [7.7, 18.6] |
|  |  |  |  |  |  |  |
| *Group 5 – Longer-term postpartum outcomes* | | | | | | |
| Weight retention > 5 kg excluded | 220 | 0.8 [-1.7, 2.7] | 216 | 6.0 [2.3, 9.6] | 292 | 10.9 [6.2, 16.0] |
| Child overweight and obesity excluded | 347 | 1.3 [-1.4, 3.6] | 304 | 7.0 [3.1, 10.9] | 444 | 12.7 [7.3, 17.4] |
| Maternal metabolic syndrome excluded | 308 | 1.0 [-1.4, 3.3] | 283 | 7.3 [3.2, 11.4] | 389 | 12.9 [7.8, 18.2] |
| Group 5 excluded | 96 | 0.9 [-1.7, 2.7] | 95 | 6.4 [2.7, 10.0] | 126 | 12.3 [6.8, 16.4] |
|  |  |  |  |  |  |  |
| *Group 5 – Longer-term postpartum outcomes* | | | | | | |
| Weight retention > 10 kg excluded | 325 | 0.9 [-1.4, 3.0] | 308 | 6.4 [2.6, 10.0] | 422 | 11.4 [6.7, 16.4] |
| Child overweight and obesity excluded | 347 | 1.3 [-1.4, 3.6] | 304 | 7.0 [3.1, 10.9] | 444 | 12.7 [7.3, 17.4] |
| Maternal metabolic syndrome excluded | 308 | 1.0 [-1.4, 3.3] | 283 | 7.3 [3.2, 11.4] | 389 | 12.9 [7.8, 18.2] |
| Group 5b excluded | 137 | 0.9 [-1.4, 2.7] | 124 | 6.7 [3.1, 10.0] | 174 | 12.3 [7.7, 16.8] |

Notes: GWG: Gestational weight gain (kg).

*Sample sizes for visits 1 and 2 vary due to the selection of weeks, missing data, and outliers of GWG in each visit.


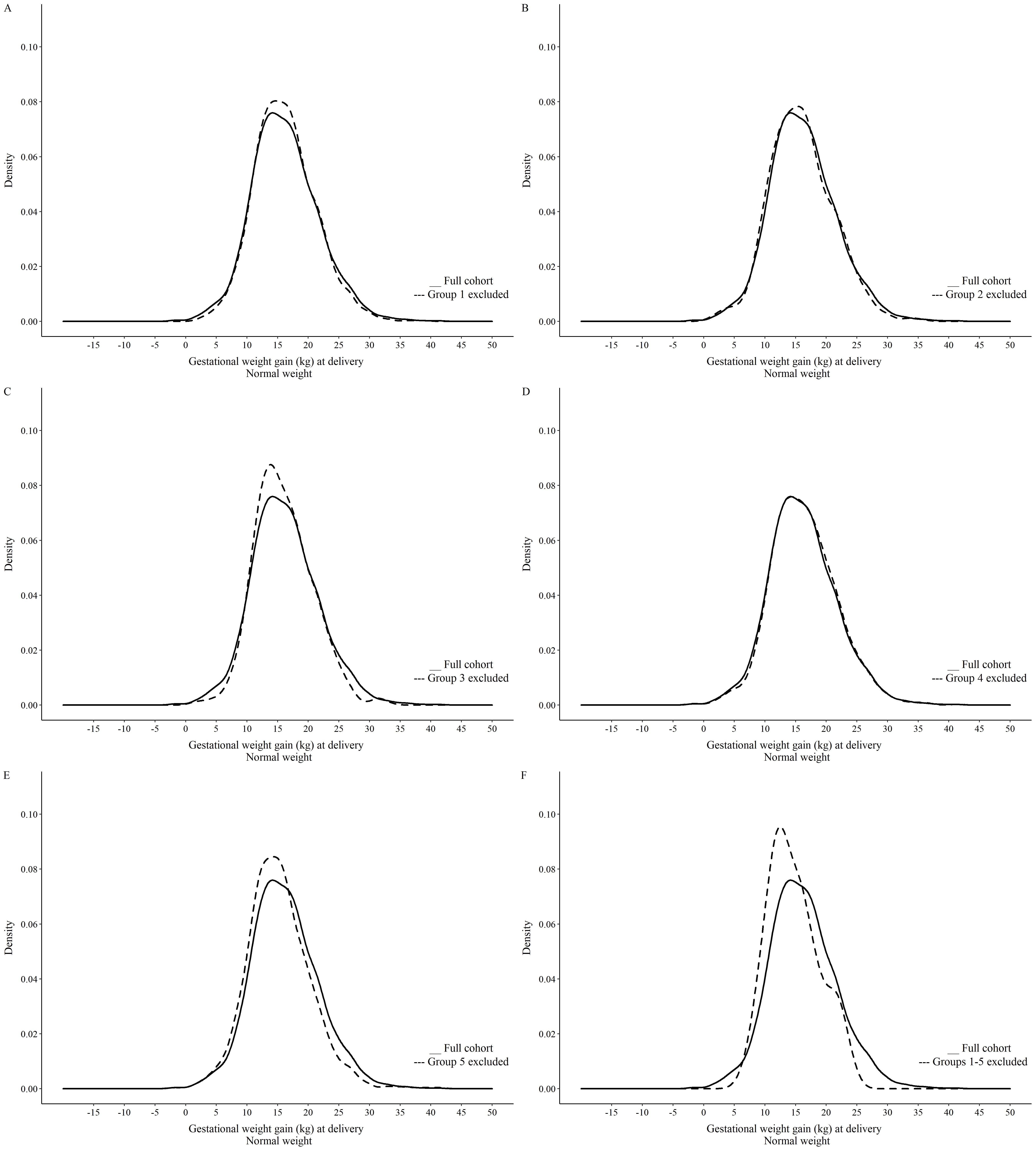


# **Supplementary Figure 3.** Distribution of gestational weight gain at delivery (26 – 42 weeks) in the full cohort and after the exclusion of the participants with each of group of conditions – individuals with pre-pregnancy normal weight.

Notes: Group 1: Socioeconomic and demographic variables, Group 2: Pre-existing comorbidities and maternal pregnancy complications, Group 3: Maternal lifestyle and behaviour factors, Group 4: Adverse neonatal outcomes, Group 5: Longer-term postpartum outcomes.


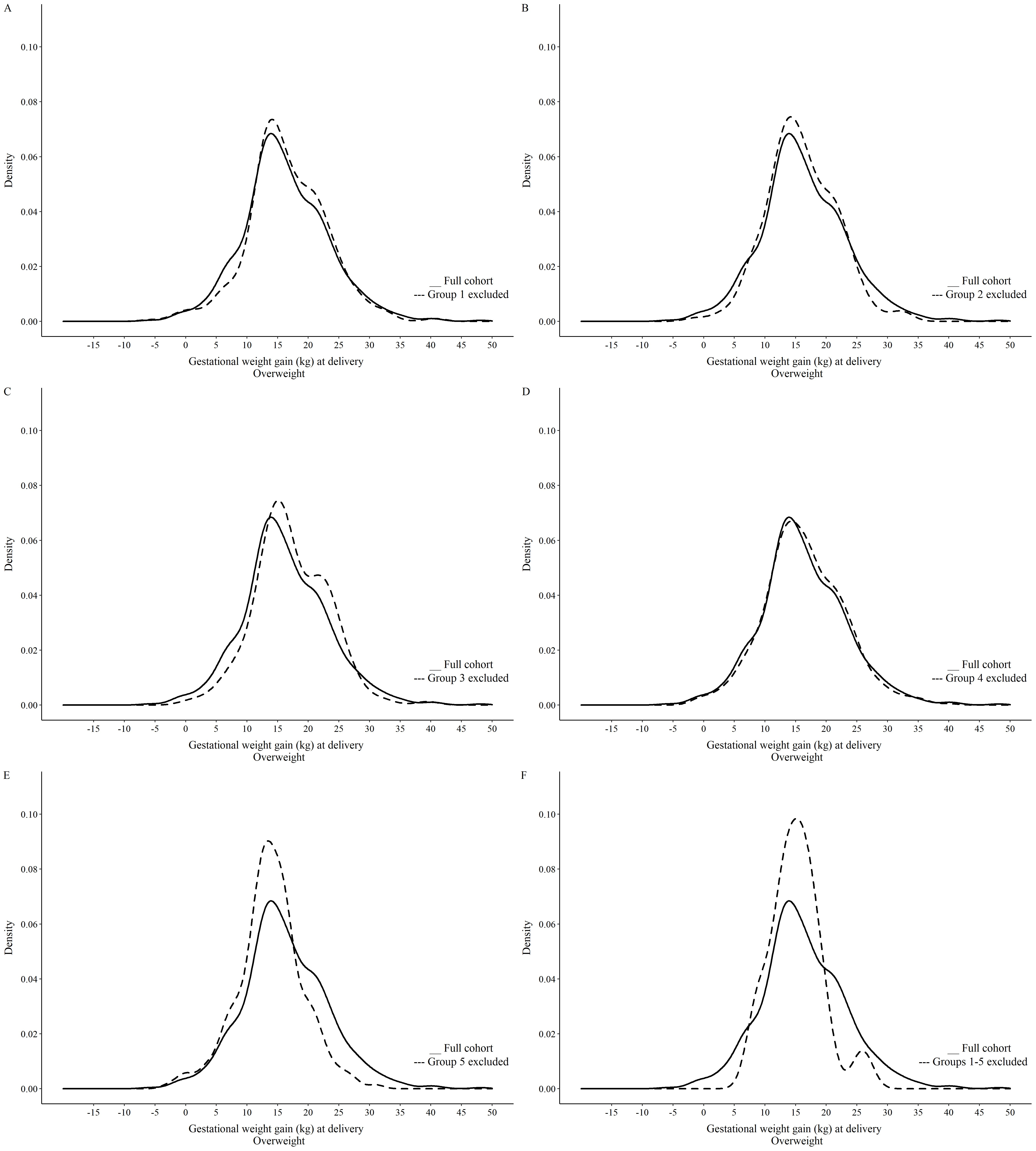


# **Supplementary Figure 4.** Distribution of gestational weight gain at delivery (26 – 42 weeks) in the full cohort and after the exclusion of the participants with each of group of conditions – individuals with pre-pregnancy overweight.

Notes: Figure S3F sample size = 17 individuals. Group 1: Socioeconomic and demographic variables, Group 2: Pre-existing comorbidities and maternal pregnancy complications, Group 3: Maternal lifestyle and behaviour factors, Group 4: Adverse neonatal outcomes, Group 5: Longer-term postpartum outcomes.


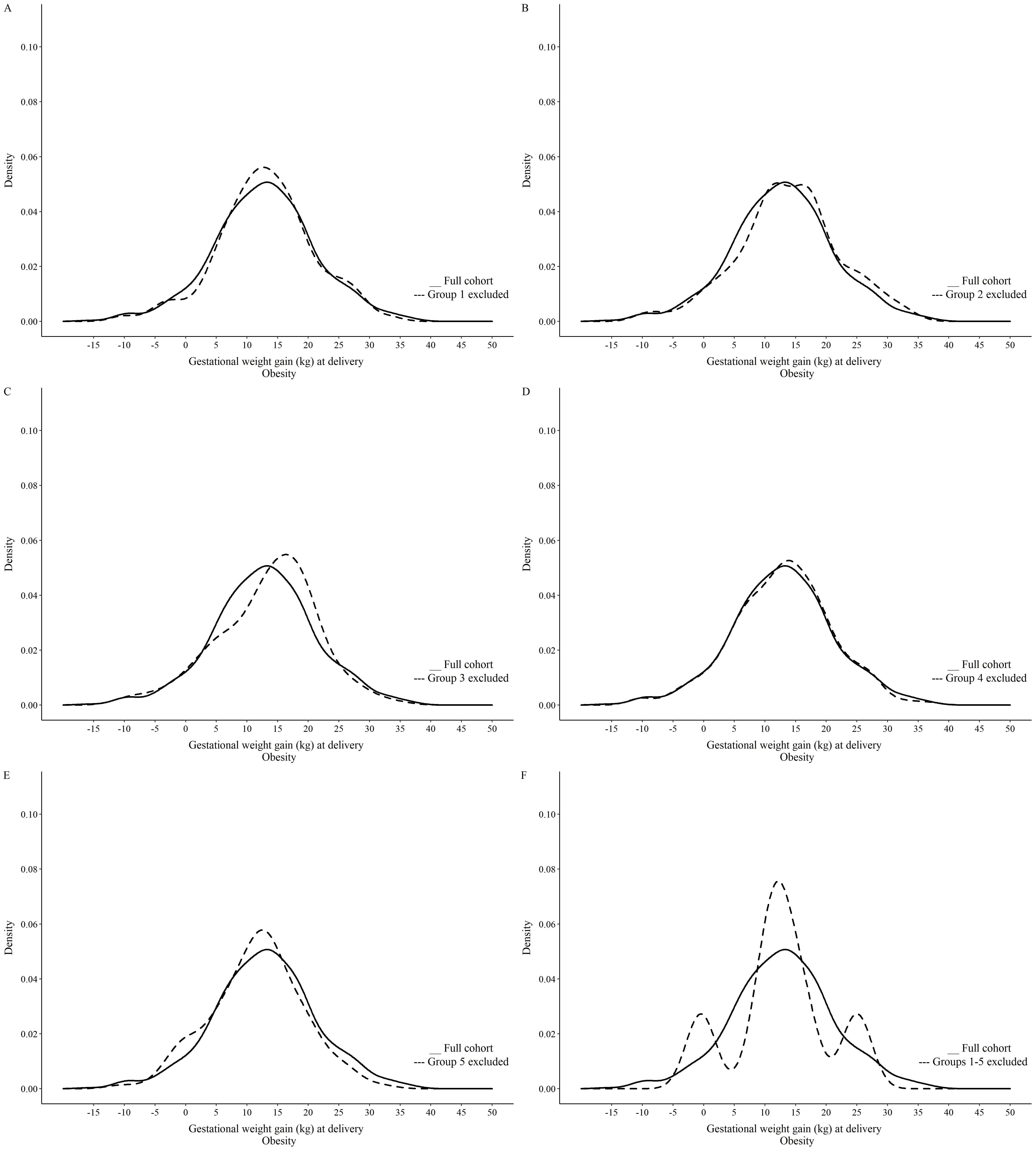


# **Supplementary Figure 5.** Distribution of gestational weight gain at delivery (26 – 42 weeks) in the full cohort and after the exclusion of the participants with each of group of conditions – individuals with pre-pregnancy obesity.

Notes: Figure S4F sample size = 6 individuals. Group 1: Socioeconomic and demographic variables, Group 2: Pre-existing comorbidities and maternal pregnancy complications, Group 3: Maternal lifestyle and behaviour factors, Group 4: Adverse neonatal outcomes, Group 5: Longer-term postpartum outcomes.
